# Supplementary material for: Asymmetric Synthesis of Quaternary Hydantoins via a Palladium-Catalyzed Aza-Heck Cyclization
Source: J Am Chem Soc. 2025 Nov 14;147(49):44692–8. doi: 10.1021/jacs.5c16022 (PMC12703750; doi:10.1021/jacs.5c16022)
Supplement: Supplementary file 2 [file ja5c16022_si_002.zip › All NMR FID Files/S57/S57_ProtonNMR/1/pdata/1/email_MLM03075C1-1_1_1.pdf]

qPROTON CDCl3 /opt/nmrdata mlmurr 18

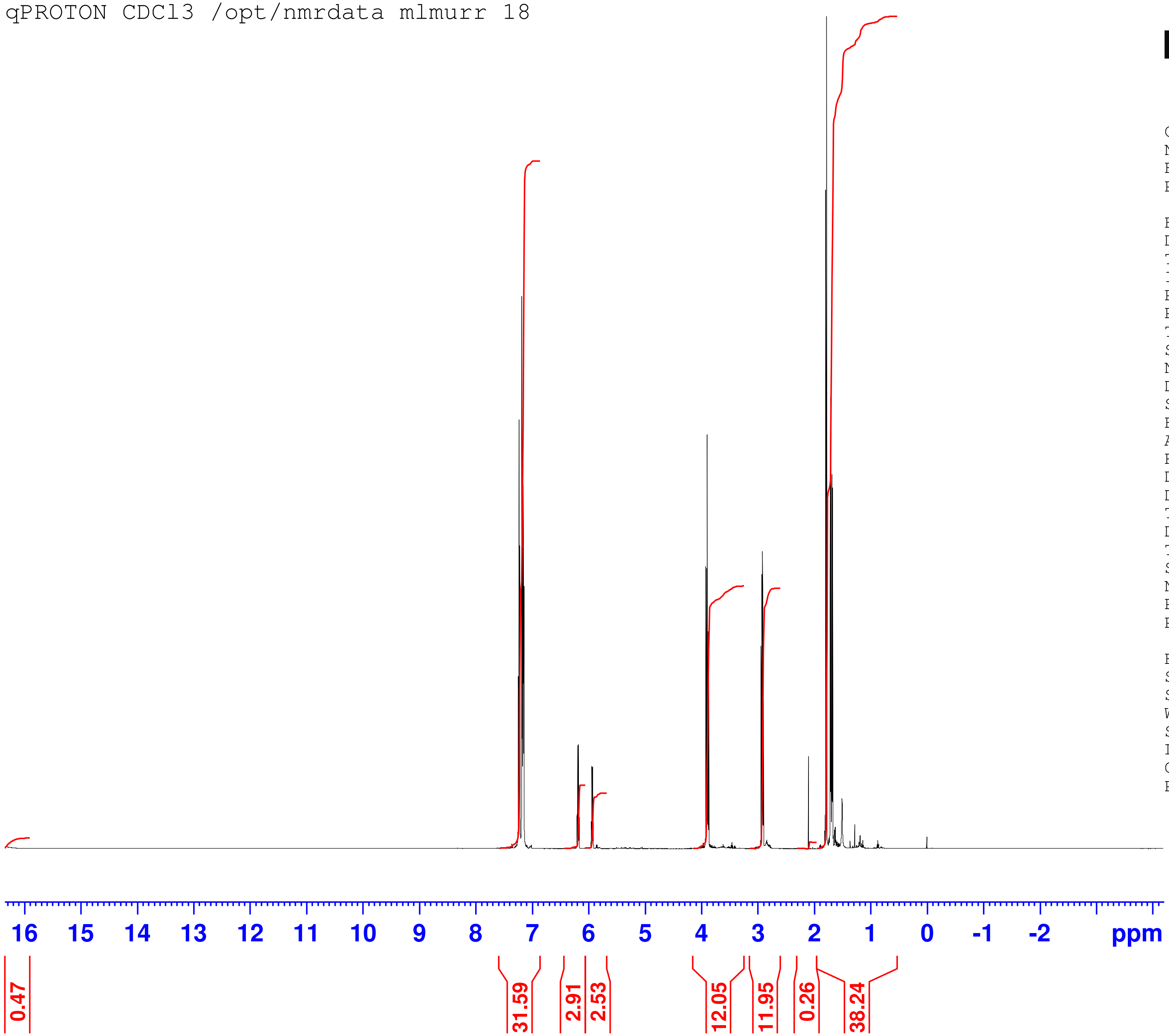

BRUKER

Current Data Parameters
NAME MLM03075C1-1
EXPNO 1
PROCNO 1

F2 - Acquisition Parameters
Date_ 20240614
Time 15.47 h
INSTRUM spect
PROBHD Z168773_0053 (
PULPROG zg
TD 65536
SOLVENT CDCl3
NS 16
DS 2
SWH 12335.526 Hz
FIDRES 0.376450 Hz
AQ 2.6563926 sec
RG 32
DW 40.533 usec
DE 18.00 usec
TE 298.0 K
D1 10.00000000 sec
TD0 1
SFO1 600.3237072 MHz
NUC1 1H
P1 12.00 usec
PLW1 21.85499954 W

F2 - Processing parameters
SI 65536
SF 600.3200575 MHz
WDW EM
SSB 0
LB 0.30 Hz
GB 0
PC 1.00
